# Effects on Balance and Walking with the CoDuSe Balance Exercise Program in People with Multiple Sclerosis: A Multicenter Randomized Controlled Trial

**DOI:** 10.1155/2016/7076265

**Published:** 2016-11-30

**Authors:** Anette Forsberg, Lena von Koch, Ylva Nilsagård

**Affiliations:** ^1^University Health Care Research Center, Faculty of Medicine and Health, Örebro University, Örebro, Sweden; ^2^Department of Neurobiology, Care Sciences and Society, Karolinska Institutet, Stockholm, Sweden

## Abstract

*Background*. Balance and walking impairments are frequent in people with multiple sclerosis (MS).* Objective*. The aim was to investigate the effects of a group-based balance exercise program targeting core stability, dual tasking, and sensory strategies (CoDuSe) on balance, postural sway, walking, perceived walking limitations, and balance confidence.* Design*. A single-blinded randomized multicenter trial. No intervention was given to controls.* Participants*. People with MS able to walk 100 meters but unable to maintain tandem stance ≥30 seconds. Eighty-seven participants were randomized to intervention or control.* Intervention*. The 60-minute CoDuSe group program, twice weekly for seven weeks, supervised by physical therapists.* Measurements*. Primary outcome was dynamic balance (Berg Balance Scale (BBS)). Secondary outcomes were postural sway, walking (Timed-Up and Go test; Functional Gait Assessment (FGA)), MS Walking Scale, and Activities-specific Balance Confidence (ABC) Scale. Assessments were performed before and after (week 8) the intervention.* Results*. 73 participants fulfilled the study. There were significant differences between the intervention and the control groups in change in the BBS and in the secondary measures: postural sway with eyes open, FGA, MS Walking Scale, and ABC scale in favor of the intervention.* Conclusions*. The seven-week CoDuSe program improved dynamic balance more than no intervention.

## 1. Introduction 

People with multiple sclerosis (PwMS) frequently report balance and walking impairments and as a consequence being restricted in activities and in performing daily tasks [1]. Walking can be affected early on in the course of the disease [2, 3]. Several studies report that PwMS have a substantial risk of falling [4–9], and walking activities are often associated with a higher risk of falls [4, 6]. A review summarizes imbalance as decreased ability to maintain a position, slower proactive balance reactions when trying to reach, and delayed response as well as difficulty maintaining stability while being exposed to external perturbations [10]. Several factors interact in causing imbalance and walking impairment: weakness, spasticity, cerebellar ataxia, slowed somatosensory conduction, and impaired central integration as well as fatigue and impaired attention [11, 12]. Additionally, walking while performing cognitive tasks is associated with reduced gait speed and stride length [13–15] and is considered a risk of falls [16, 17].

PwMS have decreased trunk stability compared to healthy subjects [18]. In standing balance, deficits with increased trunk sway are reported [19, 20], and adding a dual task increases the postural sway [21]. Furthermore, sensory disturbances evoke difficulties in balance control in quiet standing with increased postural sway [22]. The transition between movement and an upright posture can produce imbalance with difficulties in coordinating body segments during the movement [23]. Taken together, it appears that improving balance in standing and activities should be important goals in physical therapy for PwMS.

Previous studies of balance training have had differences in content and the results have not been conclusive [24]. In a study by Cattaneo et al., 44 PwMS were randomized to either motor training, combined motor and sensory training, or no intervention [25]. Using the Berg Balance Scale (BBS) as outcome measure, significant improvements were found in favor of the combined motor and sensory training. Prosperini et al. found increased postural stability in single stance and improved walking speed after visuoproprioceptive training in a single-group study where 28 PwMS fulfilled the training [26]. In a study by Hebert et al. 38 PwMS were randomized to either a vestibular rehabilitation group, an exercise control group including endurance and stretching exercises, or a control group [27]. That study found significant improvement in upright postural control in favor of the vestibular rehabilitation.

Based on these findings and in collaboration with a network of clinically based physical therapists, we developed a group-based balance exercise program. The aim of the program was to target factors of importance in maintaining balance during activities; trunk stability, dual tasking, and sensory strategies. As activation of trunk muscles is of importance for balance, the main focus of the program was on core stability exercises. The choice of exercises was inspired by Freeman et al. [28]. In a first single-group analysis of the effects of the core stability, dual tasking, and sensory strategies program (CoDuSe) on fall reduction, we found that the intervention significantly reduced both the number of falls and the proportion of fallers between the preintervention and postintervention periods [29]. Our next hypothesis was that the CoDuSe program would improve balance compared to no intervention. Thus, the aim of the present study was to investigate the effects of this seven-week, twice weekly, group-based balance exercise program that targeted core stability, dual tasking, and sensory strategies (CoDuSe) on performance in dynamic balance, postural sway, walking, perceived limitations in walking, and balance confidence.

## 2. Methods

### 2.1. Design Overview

The study design was a single-blinded two-arm (group-based balance exercises versus control) randomized controlled multicenter trial. Intervention and data collection were conducted from August 2012 to June 2013 at seven physical therapy departments in hospitals or primary health care centers in five county councils in Sweden: University Hospital in Region Örebro County; hospitals in Eskilstuna, Nyköping, Karlstad, and Västerås; and primary health care centers in Linköping and Mjölby. The study was registered in the Clinical Trials database (NCT 01582126) and was approved by the Regional Ethics Committee, Uppsala-Örebro (ID 2012/117).

### 2.2. Setting and Participants

PwMS diagnosed [30] by a neurologist were invited to participate. Further inclusion criteria were being able to walk 100 meters (use of assistive walking device was allowed) and able to get up from the floor with minor support (hence being able to participate in the intervention) but being unable to maintain tandem stance heel-toe with arms alongside the body during 30 seconds (study-specific test of balance impairment). The exclusion criteria were cognitive or linguistic difficulties that prohibited filling in the self-report instruments. The study-specific tandem stance test corresponds to one of the items in the BBS.

**Figure 1:**
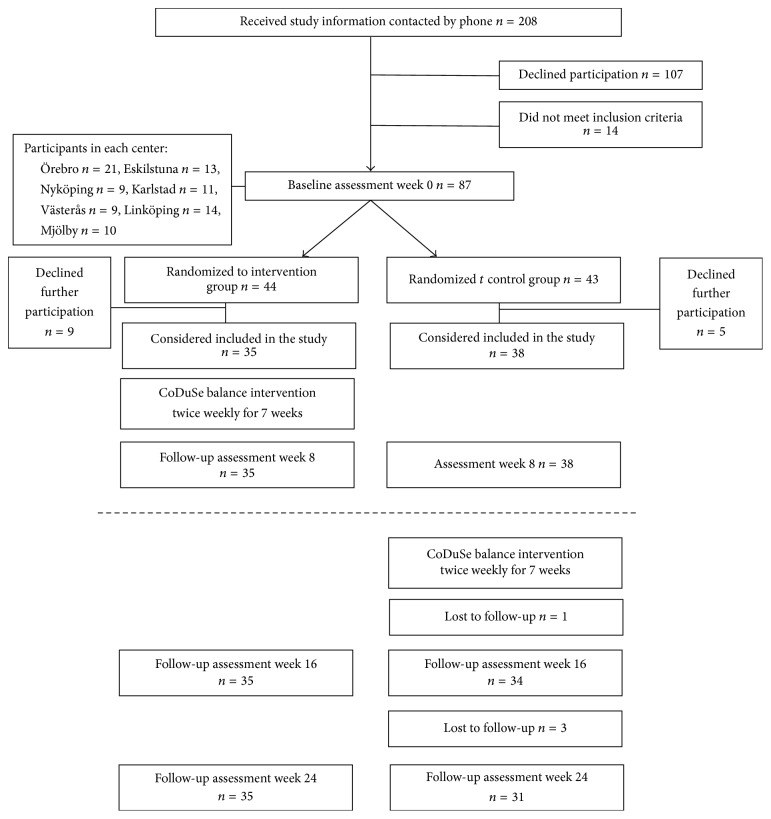
Flow chart of number of participants trough the study. Follow-up assessments at weeks 16 and 24 (below the marked line) are not presented in this study.

Possible participants were identified in the records at the participating centers or were known by the physical therapists at the centers. Eligible PwMS were given written and verbal information about the study. After one week, they were contacted and asked about participation. Participation required a written consent. In all, 101 PwMS were interested in participating in the study (Figure 1). Fourteen PwMS did not meet the inclusion criteria, and subsequently 87 PwMS were included.

### 2.3. Randomization and Procedure

Directly after the baseline assessment (week 0), the participants were randomized at each center, using sealed, opaque envelopes, The centers had separate allocations schedules. An independent statistician generated the allocation sequence that was based on the number of patients that attended the different centers. Smaller physical therapy departments had blocks with fewer participants. The physical therapist responsible for the intervention at each center opened the envelopes and allocated participants to either the intervention group or the control group. Participants randomized to the control group received the same group program but with a later start, thus a waiting-list control group.

Assessments were performed before and after (week 8) intervention. Further assessments were performed at weeks 16 and 24 but will be presented elsewhere. The assessments were performed by independent assessors, research physical therapists, who were blinded until all assessments had been completed. The assessments followed a standardized protocol. To ensure coherence in the assessment, specific training was performed before start of the study. In total six research physical therapists performed the assessments, where the authors (AF, LvK, YN) performed the majority. Adverse events were reported by the physical therapists in charge in case of the group training.

### 2.4. Intervention

The development of the CoDuSe program began with reviewing literature for evidence regarding exercise interventions aimed at reducing imbalance in PwMS. The program was then developed in an interactive process in which physical therapists who participated in the study as data collectors or led the group training designed the program together with the researchers. The length of each session and the intensity and duration of the exercise program were defined in congruence with previous research and clinical experience among the physical therapists. A manual was constructed that included descriptions of the exercises in text and pictures with progression of the exercises (a copy of the manual is available upon request, contact anette.forsberg@regionorebrolan.se). In total, 14 physical therapists were involved in the interactive process and later in execution of the intervention at the centers. The physical therapists had several years of experience working in neurological rehabilitation, mean 19 years (min 7; max 42).

The standardized intervention targeted visual, somatosensory, and vestibular aspects of balance and included balance exercises in groups of 4–7 people twice weekly for seven weeks. Each session included 20 minutes of core stability exercises inspired by Freeman et al. [28], followed by 15–20 minutes of dual-task exercises and 15–20 minutes of exercises challenging different sensory strategies. Examples of core stability exercises were as follows: in supine position with knees bent, slowly slide one heel forward to straighten leg; and, in four-point kneeling position, slide one foot in a straight line away from the body and lift the leg off the floor. Examples of dual-task exercises were walking while turning one's head and juggling a balloon. Examples of sensory strategies were standing and walking on uneven surfaces and standing with eyes closed. The participants were encouraged to maintain focus on core stability during the whole session. Each session ended with 5 minutes of stretching and relaxing.

### 2.5. Outcomes and Follow-Up

At the baseline assessment, information was collected on demographic characteristics, type of MS, use of assistive walking devices indoors and outdoors, and the number of falls during the previous 2 months. A fall was defined as an unexpected event in which the participant comes to rest on the ground, floor, or lower level [31]. To describe the participants' cognitive and physical functioning, the Symbol Digit Modalities Test (SDMT) and the Multiple Sclerosis Impact Scale (MSIS) were used. The SDMT is regarded as a measure of processing speed in the visual modality and is recommended in MS [32, 33]. For 90 seconds the participants were asked to orally express the digits associated with corresponding symbols. The MSIS [34] is a self-report measure of the impact of MS on daily functioning, composed of 20 items of physical symptoms and 9 items of psychological symptoms. Items are scored from 1 (not at all) to 5 (extremely). A higher score indicates larger perceived impact of MS.

The primary and secondary measures were performed at all assessments according to the standardized protocol. The primary outcome measure was the BBS, a measure that includes 14 items of static and dynamic balance [35]. Items are rated 0–4, with a maximum of 56. The BBS is recommended for use in both research and clinical practice in MS [36] and is considered reliable [37] and valid [38]. In samples of individuals with MS similar to this study, a minimal detectable change (MDC) of 2–4 points has been suggested [39–41]. To our knowledge, minimal clinically important difference (MCID) is not known for ambulatory people with MS.

Secondary measures included tests of postural sway, dynamic balance, walking, and perceived limitations in walking and balance confidence. Postural sway was assessed with a Swaymeter device [42] with the subject barefoot, in four different conditions: eyes open and closed on hard floor and soft cushion, respectively. The Swaymeter consists of a 40 cm long rod mounted on a 20 cm metal plate that is held over the participant's lower back by a firm belt. In the other end of the rod, a pen is vertically mounted. The participant's postural sway was recorded on a sheet of paper for each condition during 30 seconds. The sway area was calculated as the anteroposterior displacement × mediolateral displacement in millimeters [43]. Psychometric properties, including MDC and MCID, for postural sway have not been studied in MS.

Dynamic balance was assessed with the Four-Square Step Test (FSST) [44] and a 10-repetition sit-to-stand test [45]. In the FSST, the participant steps over 2.5 cm high sticks that are placed in a cross formation [44]. Participants are timed as they walk clockwise over the sticks and then counterclockwise, forward, sideways, backwards, and sideways again. The best time of two attempts was used. Assistive devices such as crutches and canes were allowed. The FSST is valid and reliable in MS, and MDC of 4.6 seconds has been presented in individuals with mild-moderate MS [46]. The sit-to-stand test is a valid measure of functional muscle strength in the lower extremities in MS [45, 47]. In this test, participants are timed as they repeat the sit-to-stand movement 10 times from an armchair. For the 5-repetition sit-to-stand test, a change of 25% has been suggested as a real change in people with MS [48].

Walking was assessed with the Timed-Up and Go (TUG) test and the Functional Gait Assessment (FGA). In the TUG test the participant is timed as she or he rises from a chair, walks 3 meters, turns around, walks back, and sits down again [49]. Use of assistive devices was allowed and the first attempt after a pretrial was used. The TUG test is a valid and reliable measure in MS [37, 38]. MDC of 10.6 seconds together with standard error of measurement of 3.81 seconds was suggested in a study including individuals with Extended Disability Status Score (EDSS) of 5.0–6.5 [37]. The TUG test was repeated with the addition of a cognitive component, the TUG^cognitive^ test, in which the participant was asked to count backwards by 3's from a random number between 20 and 100 [50]. The FGA includes 10 items covering quality of movement, deviation from intended the pathway, need of walking device, and time to perform walking activities [51]. Items are graded between 0 (severe impairment) and 3 (normal performance), giving a maximum score of 30. A Swedish validated version was used [52, 53]. In older adults MCID of 4 points has been presented [54]. To the best of our knowledge, MDC or MCID has not been estimated in ambulatory individuals with mild to moderate MS for the TUG test, the TUG^cognitive^ test, or the FGA.

Perceived limitations in walking and balance confidence were assessed with two patient-reported outcome measures, the 12-item MS Walking Scale (MSWS-12) [55] and the Activities-specific Balance Confidence (ABC) scale [56]. The MSWS-12 consists of 12 items rated from 1 (not at all limited) to 5 (extremely limited). Scores were added giving a maximum of 60. In individuals with EDSS 0–6.5, a 10% change has been regarded as an important change [57]. The ABC scale consists of 16 items describing balance-demanding activities, indoors and outdoors. The sum score ranges between 0 (no confidence) and 100 (completely confident). Validated Swedish versions were used for both the MSWS-12 [58] and the ABC scale [59]. For the ABC scale, MDC of 6.8 points has been presented for ambulatory people with stroke [60]; however, for individuals with MS values for MDC or MCID has not been presented.

### 2.6. Sample Size

Sample size was calculated based on an expected clinically significant difference between intervention group and control group of 5 points on the primary outcome measure, the BBS, and a standard deviation of 7 points [25, 37]. With alpha level at* p* = 0.05 and 80% power, it was estimated that a sample size of 32 participants had to be recruited into each group. In the calculation of sample size, the BBS was used as a continuous variable. To account for that and for a 10% possible dropout rate, we aimed to enroll 70 individuals.

### 2.7. Statistical Analysis

Mean, standard deviation (SD), range, and percentages were used to present demographic and background characteristics. Per protocol analysis was performed since several participants declined further participation shortly after the randomization (before start of exercise) due to insufficient practical information (i.e., the time for group sessions did not fit their schedules). Analysis of covariance adjusted for baseline value was performed for differences between groups with respect to change from baseline to week 8 in various assessments. Least-Square (LS) means with their confidence intervals and associated *p* values were presented from these analyses. Probability values computed by Student's *t*-test are given for changes from baseline to follow-up week 8. A *p* value of <0.05 was set as significant. SPSS version 22 and SAS System version 9.4 (SAS Institute, Cary, NC) were used to perform statistical analyses.

## 3. Results

Nine persons randomized to the intervention group declined further participation; that is, they did not begin with the group exercise sessions (Figure 1). Five persons randomized to the control group declined further participation. The study sample that performed both the pre- and postintervention assessments consisted of 73 participants; intervention group (*n* = 35) and control group (*n* = 38). Demographic and background characteristics are presented in Table 1. Characteristics of the 14 persons who declined further participation after the baseline assessment were similar overall to the participants included in the study analyses (mean age 51 years, mean 14 years since MS diagnosis, women 71%, relapsing–remitting MS 50%, and assistive device indoors 21% and outdoors 57%).

The group balance exercise sessions were well attended, with median 12 sessions in the intervention group (Table 1). Two adverse events were reported: one participant lost balance during challenging tasks in standing and fell on a soft carpet, and one fell while standing on his/her knees. No injuries were reported.

Results for the outcome measures before and after intervention and differences between groups are presented in Table 2. There was a significant difference between the groups in change in the primary outcome measure, the BBS, between baseline and week 8 in favor of the intervention. There were also significant differences in change in the secondary measures postural sway eyes open on hard floor and soft cushion, the FGA, the MSWS-12, and the ABC scale in favor of the intervention.

## 4. Discussion

After seven weeks of training with the CoDuSe exercise program, a significant difference in favor of the intervention was found for the primary outcome, the BBS. On some of the secondary measures, significant improvements were found also in favor of the intervention: postural sway with eyes open, FGA, MSWS-12, and ABC scale. Earlier we have reported a reduced number of falls and proportion of fallers after the 7-week intervention [29].

### 4.1. Strengths of the Trial

The CoDuSe program was feasible to perform in the clinical settings. The exercise sessions were well attended and few adverse events took place in spite of the fact that the exercises were experienced as challenging. Significant improvements were found on the patient-rated outcome measures measuring balance confidence and experiences of walking limitations. These findings suggest that the CoDuSe program can be a way to address walking and activity limitations related to imbalance.

Since balance consists of a mix of components, exercises that aim to improve balance can target different functions, strength, and endurance but should also challenge the participants' postural control and balance while performing activities. The physical therapists in charge of the exercise groups reported that the exercises targeted balance well and could be individualized within the frame of the program.

The intervention was developed in an iterative process in which all the physical therapists engaged in the study took an active part. Before start of the study the physical therapists tried the exercises, and at the subsequent meeting their experiences were the basis for a consensus discussion of which exercises were suitable and possible to perform at all the participating centers. However, with multiple settings there is a risk for deviations from the intervention manual, and to ensure fidelity to the CoDuSe manual the authors had frequent contact on phone and e-mail with the centers.

Inclusion criteria and outcome measures were chosen in dialogue with the participating physical therapy departments to ensure that the measures could be performed with portable equipment in the participating clinics. The study-specific inclusion criteria of heel-toe tandem stance was chosen as a quick test of balance impairment, but it also mirrors the test protocol in that one of the items of the BBS was used, however slightly modified. Adherence with the test protocol was ensured in that, before start of the study, standardized training was performed with the research physical therapists to ensure that the data collection was stringently performed.

### 4.2. Limitations of the Study

There are several limitations in this study. One of them is the choice of using a waiting-list control group. For ethical reasons we wanted to provide the intervention to all participants; therefore, a waiting-list design was considered appropriate. However, this prohibited comparison between groups at the follow-up assessments at weeks 16 and 24. Applying waiting-list designs has been suggested to overestimate treatment effects in that participants perceive that they are expected to “wait” until receiving the intervention and compliantly do so [61]. In this study the participants were instructed to continue with their usual activities; however, the level or intensity was not monitored. Yet, as reported in Table 2, the participants in the waiting-list control group did also improve in several of the outcome measures suggesting that the treatment effect is not overestimated.

Another limitation is the number of drop-outs. Recruiting participants to a group intervention with set days and times for the training was more difficult than anticipated. A total of 107 persons declined participation, several because of participating difficulty during office hours for 7 weeks. As many as 14 PwMS who had performed the baseline assessment and then were randomized declined further participation. This was mostly due to not having received clear information about the expectations and routines of the study, such as the time and days for the group training. However, this affects the generalizability of the results with only a few of the participants working full time. The experiences of this recruitment process raise the concern that only a limited number of people with chronic diseases who may benefit from training may have the possibility to attend training classes/sessions during office hours.

A third limitation is the lack of participant blinding. Unfortunately, in many physical therapy intervention studies it is not possible to mask the given intervention. In this study we could have provided control intervention addressing, for example, arm function. However, with the waiting-list design we had to present the intervention already in the information letter. There is therefore a risk that the benefit of the intervention is overestimated in the patient-reported outcome measures. On the other hand, the blinded assessors strengthen the accuracy of the performance based measures of balance and walking.

### 4.3. Explanation of Findings

There is a growing body of evidence for the benefits of exercise training for PwMS on aerobic capacity, muscular strength, fatigue, and health-rated quality of life [62]. The evidence for specific balance training has been weaker, probably due to few published studies having an explicit theoretical background in addressing visual, somatosensory, and vestibular impairments [24]. We based parts of the CoDuSe concept upon the findings of Cattaneo et al. [25] and Prosperini et al. [26]. In the study by Cattaneo et al. larger improvements were found on the BBS and the Dynamic Gait Index for combined motor and sensory strategy exercises compared to solely motor strategy exercises or nonspecific exercises. Prosperini et al. [26] focused the intervention on visuoproprioceptive exercises in double and single stance with and without an equilibrium board. Improved balance in single stance and gait speed was found. The exercise intervention provided in the study by Hebert et al. [27] was in part similar to the CoDuSe concept, with challenging dual tasks on different surfaces and sensory conditions. Improvements were reported in static balance; however, dynamic balance was not measured. With the CoDuSe exercise program, improvements were found in both postural sway and dynamic balance as well as during walking. The results in the present study and other studies [25–27] suggest that challenging exercises that promote sensory compensation seem to have an impact on dynamic and static balance and also can reduce the risk of fall [26, 29].

Another component of the CoDuSe concept was core stability. Core stability has mostly been studied in sports medicine, and it has been reported that the contributions of the various trunk muscles depend on the task being performed [63]. The trunk muscles must work coherently to achieve core stability, and the role of sensory-motor control is more important than the role of strength or endurance of the trunk muscles. Including specific core exercises in MS rehabilitation was first introduced in a series of single case studies by Freeman et al. [28]. Improvements were found for timed walking and the MSWS-12 and in forward and lateral reach. Other smaller studies have also shown that incorporating trunk exercises or Pilates exercises can be beneficial for PwMS [64, 65]. On the other hand a recent study showed that Pilates based core stability exercises had no effect on 10-meter walking, the MSWS-12, or the ABC scale compared to relaxation exercises [66]. In the beginning of the intervention period in the present study focus was on activating the deep abdominal muscles in neutral spinal alignment. As the exercises progressed, the physical therapist facilitated, verbally or hands-on, keeping the abdominal muscles activated. The deep abdominal muscles have been found to assist in stabilization of the spine and to play a role in postural control [67]. An explanation of the improvements on balance found in the present study could be that focus on core stability was maintained during the challenging exercises in sitting, standing, and walking.

Differences in favor of the intervention were found also on some of the secondary measures. The FGA includes different balance components such as walking without visual support, walking with narrow base of support, changing speed, raising one's foot, and climbing over an obstacle. Several of these components were targeted by the CoDuSe intervention. Significant improvements were also found for the MSWS-12 and the ABC scale as well as postural sway with eyes open. However, changes found in the present study were sometimes smaller than known MDC or the suggested clinically important changes. Nevertheless, the obtained changes on the BBS and the ABC scale can be regarded as real changes exceeding MDC.

### 4.4. Future Research

This study population consisted of people with mild-moderate MS able to walk 100 meters, and the results may only be applicable to people with this level of balance and walking impairment. Further studies should investigate the appropriateness of the exercise concept for people with more severe balance and walking impairments. The number of web-based exercise programs is growing and a further development of the CoDuSe concept is to transfer the program into an interactive exercise module. This could increase the number of persons who can take part in these exercises. However, solutions for safety during the challenging balance exercises must be considered. Outside of the study protocol, the PwMS reported that they had noticed the improved core stability and could apply it in everyday situations when they felt wobbly, thus suggesting the clinical significance of a concept targeting different balance components. This, however, needs further investigation.

## 5. Conclusions

The seven-week program targeting core stability, dual tasking, and sensory strategies improved dynamic balance measured with the BBS in people with mild-moderate MS more than no intervention. There were differences in favor of the intervention group for secondary measures of postural sway, walking, and perceived limitation of walking and balance confidence.

## Figures and Tables

**Table 1:** Demographic and background characteristics.

Variable	Intervention group (*n* = 35)	Control group (*n* = 38)
	Mean (SD) [rang]	Mean (SD) [range]

Age in years	52 (10) [28–75]	56.3 (11) [29–75]
Years since diagnosed with MS	15 (9) [5–14]	16 (11) [1–46]
Multiple Sclerosis Impact Scale (MSIS) physical subscale (0–100)	54 (18) [29–90]	56 (14) [26–80]
Multiple Sclerosis Impact Scale (MSIS) psychosocial subscale (0–100)	22 (10) [10–45]	22 (8) [9–41]
Symbol Digit Modalities Test, number of correct digits	39 (14) [12–73]	43 (13) [12–67]
Group exercise sessions, attendance	12.0 (2.1) [5–14]	

	Numbers (%)	Numbers (%)

Women	28 (80)	31 (82)
Type of MS		
Relapsing-remitting	20 (57)	13 (34)
Primary progressive	4 (11)	5 (13)
Secondary progressive	11 (31)	20 (53)
Assistive walking device indoors	5 (14)	7 (18)
Assistive walking devices outdoors (including wheel-chair/scooter)	18 (51)	26 (68)
Other illnesses	9 (20)	9 (21)
Working		
Part time, part sick leave	10 (29)	11 (29)
Full time	2 (6)	1 (3)
Full time, sick leave	16 (46)	15 (40)
Retired due to age	5 (14)	11 (29)
Unemployed	2 (6)	1 (3)

**Table 2:** Results on the outcome measures at baseline and postintervention (week 8) assessments, change from baseline, and differences between groups.

Variable (score range)	Intervention group *n* = 35		Control group *n* = 38		Difference between groups
	Baseline week 0 mean (SD)	Change from week 0 to week 8 Mean (SD), *p* value	Baseline week 0 mean (SD)	Change from week 0 to week 8 mean (SD), *p* value	LS Means for change from week 0 to week 8 mean (95% confidence interval), *p* value
Berg Balance Scale (0–56)	48.9 (5.8)	2.6 (4.1) *p* < 0.001	45.1 (9.0)	1.6 (4.1) *p* = 0.020	2.1 (0.5; 3.8) *p* = 0.011
Postural sway area, eyes open on hard floor (mm^2^)	1303 (1612)	−423 (1789) *p* = 0.17	1438 (1322)	506 (1877) *p* = 0.11	−997 (−1788; −206) *p* = 0.014
Postural sway area, eyes closed on hard floor (mm^2^)	2190 (2413)	−737 (2662) *p* = 0.12	4020 (5918)	−100 (4308) *p* = 0.89	−1036 (−2718; 645) *p* = 0.22
Postural sway area, eyes open on soft cushion (mm^2^)	3909 (3417)	−1230 (2906) *p* = 0.017	4096 (3728)	1133 (4500) *p* = 0.17	−2022 (−3608; −436) *p* = 0.013
Postural sway area, eyes closed on soft cushion (mm^2^)	8068 (5586)	−1040 (5632) *p* = 0.40	9477 (6623)	−2544 (7382) *p* = 0.12	−322 (−3517; 2874) *p* = 0.84
FSST (s)	19.9 (11.9)	−0.5 (11.0) *p* = 0.77	30.3 (28.0)	−3.5 (10.0) *p* = 0.055	1.7 (−3.3; 6.6) *p* = 0.51
Sit-to-stand (s)	35.2 (12.1)	−3.6 (8.2) *p* = 0.013	42.0 (16.6)	−4.1 (9.8) *p *= 0.014	−2.2 (−5.6; 1.2) *p* = 0.21
TUG test (s)	13.7 (5.5)	0.5 (8.5) *p* = 0.74	17.0 (9.1)	−1.0 (3.8) *p* = 0.13	1.4 (−1.7; 4.5) *p* = 0.37
TUG_cog_ test (s)	17.6 (8.3)	−0.1 (12.4) *p* = 0.94	24.0 (16.4)	−3.9 (8.9) *p* = 0.011	1.3 (−3.3; 5.9) *p* = 0.58
FGA (0–30)	15.8 (5.4)	2.7 (4.2) *p* < 0.001	14.6 (4.7)	0.7 (2.0) *p* = 0.037	2.1 (0.6; 3.6) *p* = 0.0079
MS walking scale (12–60)	40.0 (9.9)	−3.4 (5.0) *p* < 0.001	41.6 (9.7)	0.1 (5.2) *p* = 0.88	−3.7 (−6.0; −1.3) *p* = 0.0026
ABC scale (0–100)	59.3 (22.6)	7.7 (16.0) *p* = 0.0074	58.6 (19.8)	−1.9 (13.2) *p* = 0.37	9.9 (4.4; 15.4) *p* < 0.001

Mean (SD) is given for baseline week 0; mean (SD) and *p* value computed by Student's *t*-test are given for change from baseline to follow-up week 8. Mean (95% confidence interval) and *p* value computed by using analysis of covariance (ANCOVA) adjusted for baseline value are given for difference between groups.

LS Means: Least Square Means adjusted for baseline value obtained from ANCOVA.

FSST: Four-Square Step Test; TUG test: Timed-Up and Go test; TUG_cog_ test: TUG test with a cognitive task; FGA: Functional Gait Assessment; ABC scale: Activities-specific Balance Confidence scale.
